# Identification of MARK2, CCDC71, GATA2, and KLRC3 as candidate diagnostic genes and potential therapeutic targets for repeated implantation failure with antiphospholipid syndrome by integrated bioinformatics analysis and machine learning

**DOI:** 10.3389/fimmu.2023.1126103

**Published:** 2023-10-13

**Authors:** Manli Zhang, Ting Ge, Yunian Zhang, Xiaolin La

**Affiliations:** ^1^Center for Reproductive Medicine, First Affiliated Hospital of Xinjiang Medical University, Urumqi, China; ^2^Basic Medical College of Xinjiang Medical University, Urumqi, China; ^3^State Key Laboratory of Pathogenesis, Prevention, and Treatment of High Incidence Diseases in Central Asia, Xinjiang Medical University, Urumqi, China

**Keywords:** repeated implantation failure, anti-phospholipid syndrome, bioinformatics analyses, nomogram, immune infiltration, machine learning

## Abstract

**Background:**

Antiphospholipid syndrome (APS) is a group of clinical syndromes of thrombosis or adverse pregnancy outcomes caused by antiphospholipid antibodies, which increase the incidence of *in vitro* fertilization failure in patients with infertility. However, the common mechanism of repeated implantation failure (RIF) with APS is unclear. This study aimed to search for potential diagnostic genes and potential therapeutic targets for RIF with APS.

**Methods:**

To obtain differentially expressed genes (DEGs), we downloaded the APS and RIF datasets separately from the public Gene Expression Omnibus database and performed differential expression analysis. We then identified the common DEGs of APS and RIF. Gene Ontology and Kyoto Encyclopedia of Genes and Genomes pathway enrichment analyses were performed, and we then generated protein-protein interaction. Furthermore, immune infiltration was investigated by using the CIBERSORT algorithm on the APS and RIF datasets. LASSO regression analysis was used to screen for candidate diagnostic genes. To evaluate the diagnostic value, we developed a nomogram and validated it with receiver operating characteristic curves, then analyzed these genes in the Comparative Toxicogenomics Database. Finally, the Drug Gene Interaction Database was searched for potential therapeutic drugs, and the interactions between drugs, genes, and immune cells were depicted with a Sankey diagram.

**Results:**

There were 11 common DEGs identified: four downregulated and seven upregulated. The common DEG analysis suggested that an imbalance of immune system-related cells and molecules may be a common feature in the pathophysiology of APS and RIF. Following validation, MARK2, CCDC71, GATA2, and KLRC3 were identified as candidate diagnostic genes. Finally, Acetaminophen and Fasudil were predicted as two candidate drugs.

**Conclusion:**

Four immune-associated candidate diagnostic genes (MARK2, CCDC71, GATA2, and KLRC3) were identified, and a nomogram for RIF with APS diagnosis was developed. Our findings may aid in the investigation of potential biological mechanisms linking APS and RIF, as well as potential targets for diagnosis and treatment.

## 1 Introduction

Repeated implantation failure (RIF), which accounts for 5% to 10% of assisted reproductive technology (ART) failures (1), is a syndrome where patients with infertility continue to fail to achieve a clinical pregnancy despite several *in vitro* fertilization-embryo transfers (IVF-ET), a process with a cumulative live birth rate reaching 34.7% (2). Although the diagnostic criteria for RIF have not yet been standardized, clinical studies have defined RIF as the inability of women under the age of 40 to achieve clinical pregnancy following the transfer of at least four high-quality embryos in at least three fresh or frozen cycles (3). RIF has a complicated etiology that includes anatomical factors of the reproductive organs, chromosomal abnormalities of the couple or embryos, endocrine abnormalities, and infections (4). Although the etiology of some RIF patients has not yet been identified, it is believed to be connected to immunological factors (5). It has become evident that RIF is one of the most important issues in the assisted reproduction field because of its complicated etiology and the significant psychological burden it places on patients and their partners.

Antiphospholipid syndrome (APS) is a serious autoimmune disease that is characterized by persistent positivity for anti-phospholipid antibodies (APLs), including lupus anticoagulant (LA), anticardiolipin antibody (ACL), and anti-β2-glycoprotein 1 (β2-GP1) antibody. APS can cause microthrombosis of the chorionic plate at the maternal-fetal interface, which can lead to recurrent miscarriage, placental malfunction, preterm pre-eclampsia, fetal growth restriction, fetal distress, and even stillbirth (6). According to studies (7), 5% to 20% of women of childbearing age exhibit clinical symptoms of APS. If patients with positive APLs are not treated with the proper interventions or therapies, the pregnancy loss rate can reach 24% to 60%. APL positivity is linked to IVF-ET failure and correlates strongly with RIF (8). IVF failure and pregnancy complications were more common in APL-positive patients than in negative patients (9). Patients with RIF had higher rates of positive ACL antibodies, anti-β2-GP1 antibodies, and anti-phosphatidylethanolamine antibodies. The IVF-ET failure rate was significantly higher in triple-positive or more APL subtype-positive patients than in normal people (10). These findings imply that infertility patients with APS are at risk of RIF.

Multiple mechanisms can trigger T cell activation and the accompanying cytokine production in APS patients *in vivo*, which affect the immune system’s normal regulation and disturb immunological homeostasis (11). In addition, APL can inhibit chorionic villous cells from migrating or invading, decrease the expression levels of complement regulatory proteins, activate complement on the surface of trophoblast cells, and trigger inflammatory responses (12). Furthermore, APS patients can develop microvascular thrombosis, substantially impairing endometrial metaplasia and early embryonic implantation, which ultimately results in female infertility, unsuccessful implantation, and spontaneous abortion (13, 14). These findings strongly support a connection between APS and RIF. However, the underlying mechanisms are still poorly understood, making it necessary to investigate their shared pathophysiology and genetic details. In this study, we sought to explore the mechanisms by which APS affects immune tolerance in pregnancy, leading to embryo implantation failure, and to further establish and investigate the networks involved in inflammation and immune regulation in order to improve the diagnosis and clinical management of such patients.

**Figure 1:**
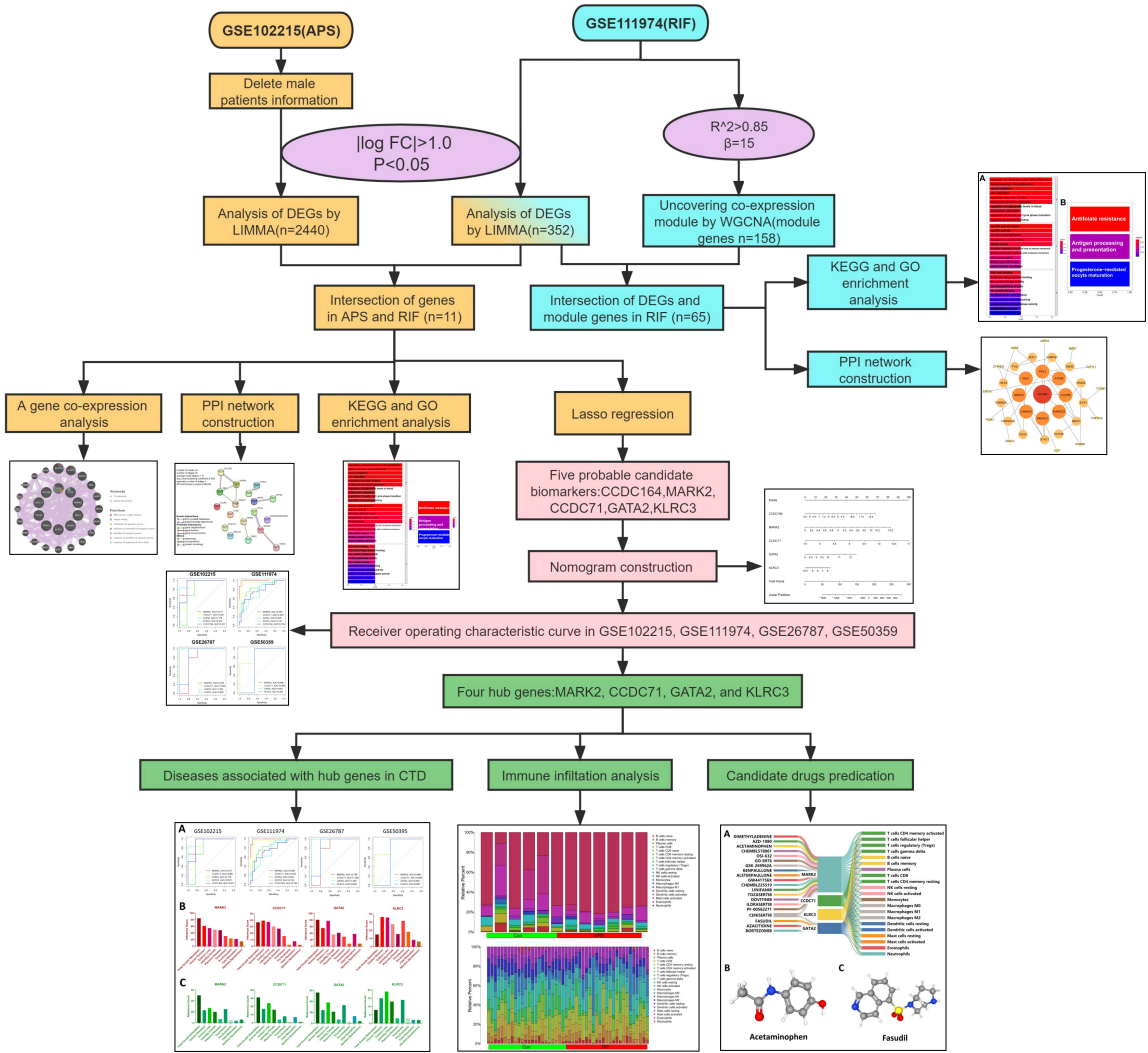
Flow chart of our research.

To examine the common pathogenesis of APS and RIF, we screened public databases for common differentially expressed genes (DEGs) between the two diseases and used them to build diagnostic models and identify potential therapeutic drugs. This is the first study to investigate common markers between APS and RIF using a systems biology approach. This work will provide new insights and direction for understanding the biological mechanisms of both diseases, which will aid in the design of dual-purpose treatment methods. **Figure 1** depicts the study flow chart for this investigation.

## 2 Materials and methods

### 2.1 Data collection and preprocessing

Microarray datasets were downloaded from the Gene Expression Omnibus (GEO) database (http://www.ncbi.nlm.nih.gov/geo/), which has a significant amount of microarray, second-generation sequencing, and other high-throughput sequencing data (15). Using the keywords “antiphospholipid syndrome” and “implantation failure”, we searched related gene expression datasets. The male specimens and non-human samples tested were eliminated. Finally, the GSE102215 (16) (based on GPL16791), GSE50395 (17) (based on GPL4133), GSE111974 (18) (based on GPL17077), and GSE26787 (19) (based on GPL570) datasets were downloaded from the GEO database.

### 2.2 Identification of common genes

For GSE102215, after excluding the male patients, the DEGs between healthy and APS samples were identified using the limma package (version 3.44.3) (20). DEGs were identified as genes that met the specific cutoff criteria of false discovery rate <0.05 and |log fold change|>1.0. For GSE111974, genes with false discovery rate <0.05 and |log fold change |>1.0 were defined as DEGs. DEG expression was visualized by volcano plots and heatmaps using the ggplot2 package (version 3.3.2) (21) and pheatmap package (version 1.0.12) (22). To screen for common DEGs associated with APS and RIF, the online Venn diagram platform (http://bioinformatics.psb.ugent.be/webtools/Venn/) was used to collect their overlapping DEGs.

### 2.3 Module gene selection of RIF by weighted gene co-expression network analysis

WGCNA is an algorithm that analyzes the gene expression patterns of multiple samples, clusters genes with similar expression patterns to form different modules, and analyzes the associations between modules and phenotypes or traits, as well as the hub genes in the network (23). To retrieve the RIF-related modules, WGCNA was employed to analyze the GSE111974 dataset. The data were reviewed to find any outliers in the samples, and all samples from the RIF dataset were properly clustered. Using the criterion of R^2^>0.85, a suitable soft-thresholding power β was calculated to determine the scale-free topology. Next, co-expression modules were generated using hierarchical clustering, and the results were presented in a hierarchical clustering tree. Automatic module merging was performed for modules with highly related trait genes (minimum number of module genes set to 30, merge cutting height = 0.25). Finally, the expression profile of each module was obtained by calculating the module eigengene and the correlations between the clinical features and module eigengene. We selected the modules that had better correlation coefficients with clinical characteristics(*P*-value<0.05), and the genes from those modules were then chosen for further analysis.

### 2.4 Gene ontology and kyoto encyclopedia of genes and genomes enrichment analysis

A dynamically updated set of controlled vocabulary is provided by GO, an internationally standardized classification system for gene functions, to comprehensively characterize the features of genes and gene products in living organisms (24). KEGG is a database that combines genomic and functional information and conducts systematic assessments of gene functions (25). The R package clusterProfiler (26) was used to perform a functional enrichment study, with a criterion of *P*-value<0.05. GO and KEGG analyses were performed twice in this work.

### 2.5 Construction of a protein-protein interaction network

Search Tool for the Retrieval of Interacting Genes (STRING; http://string-db.org) (version 11.5) is a comprehensive database for searching interactions between proteins, including direct physical interactions and indirect functional correlations between proteins (27). Using STRING with an interaction score > 0.4, the PPI networks of module genes and common DEGs were constructed. The PPI network was visualized by Cytoscape software (version 3.8.2) (28).

### 2.6 Co-expression analysis of common DEGs

To further explore the interrelation between common DEGs, the co-expression network was constructed using the GeneMANIA online tool. Using extensive genomic and proteomics data, the GeneMANIA (http://genemania.org/) (29) database allows for the construction of co-expression networks of identified common genes to recognize functionally related genes and weigh them based on expected values.

### 2.7 Machine learning

Candidate genes for RIF with APS were further filtered using the least absolute shrinkage and selection operator (LASSO) regression. While fitting a generalized linear model, LASSO regression is characterized by variable selection and complexity regularization, preventing overfitting and improving the predictive accuracy and comprehensibility of the clinical prediction models (30). LASSO regression was performed using the glmnet R packages (31). The genes screened by LASSO regression were considered candidate hub genes in RIF with APS diagnosis.

### 2.8 Nomogram construction and evaluation

The construction of nomograms is valuable in diagnosing clinical RIF with APS. Using the candidate hub genes, the nomogram was developed using the rms R package (32). “Points” denotes the score of candidate hub genes, and “Total Points” represents the sum of all the above gene scores. To evaluate the diagnostic value of candidate hub genes for RIF and APS, receiver operating characteristic (ROC) curves were subsequently constructed using the pROC R package, and the area under the ROC curve (AUC) was calculated separately (33). AUC>0.75 was considered an ideal diagnostic value. Genes with better diagnostic performance were screened for drug prediction.

### 2.9 Immune cell infiltration analysis

The CIBERSORT is a computational method that uses tissue gene expression profiles to identify the proportions of different immune cells (34). Immune cell infiltration analysis in controls versus APS and RIF samples was conducted with the Cibersort R package. Bar graphs were used to display the proportions of different immune cell types, and violin plots were used to compare these proportions between the APS and control groups, as well as the RIF and control groups. Correlation heatmaps were plotted with the corrplot R package (35) to show the correlations between the immune cells and candidate diagnostic genes.

### 2.10 Hub gene interactions with diseases and prediction of candidate drugs

The Comparative Toxicogenomics Database (CTD) (http://ctdbase.org/) (36) integrates data from a variable number of genes, chemical substances, functional phenotypes, and interactions between diseases. It greatly facilitates the study of disease-related environmental exposure factors and potential mechanisms of drug action. To investigate the relationships between candidate diagnostic genes and diseases, we analyzed the inference score and reference count for candidate diagnostic genes with associated diseases using CTD. Inference scores and reference counts were visualized by histograms.

Drugs that interact with candidate diagnostic genes were obtained from the Drug Gene Interaction Database (DGIdb) (https://dgidb.genome.wustl.edu/) (37) for predicting potential drugs for the treatment of APS and RIF, and the 3D structures are displayed by the PubChem website (https://pubchem.ncbi.nlm.nih.gov/) (38). The Sankey diagram was created using SankeyMATIC (https://sankeymatic.com/), which was used to characterize the interactions between potential drugs, candidate diagnostic genes, and immune cells.

### 2.11 Statistical analysis

We conducted statistical analyses with R software (version 4.2.1) and GraphPadPrism (version 8.0.1). The Wilcoxon test was used to measure the differences in expression between the two groups. Statistics were considered significant at *P*-value<0.05.

## 3 Results

### 3.1 GEO information

Four GEO datasets (GSE102215, GSE11974, GSE50395, and GSE26787) were selected for analysis. **Table 1** summarizes the specific information of these four datasets, including the GSE number, platforms, disease, samples, source types, and groups. GSE102215 and GSE111974 were paired for the DEG analysis, and GSE50395 and GSE26787 were paired to confirm the hub genes’ diagnostic efficacy.

**Table 1:** Summary of the four Gene Expression Omnibus (GEO) datasets involving antiphospholipid syndrome (APS) and repeated implantation failure (RIF) patients.

ID	GSE number	Platform	Disease	Samples	Source types	Grope
1	GSE102215	GPL16791	APS	6 patients and 6 controls	peripheral venous blood	Discovery cohort
2	GSE50395	GPL4133	APS	3 patients and 3 controls	peripheral venous blood	Validation cohort
3	GSE111974	GPL17077	RIF	24 patients and 24 controls	Endometrium	Discovery cohort
4	GSE26787	GPL570	RIF	5 patients and 5 controls	Endometrium	Validation cohort

APS, antiphospholipid syndrome. RIF, repeated implantation failure.

### 3.2 Screening of common DEGs

**Figure 2:**
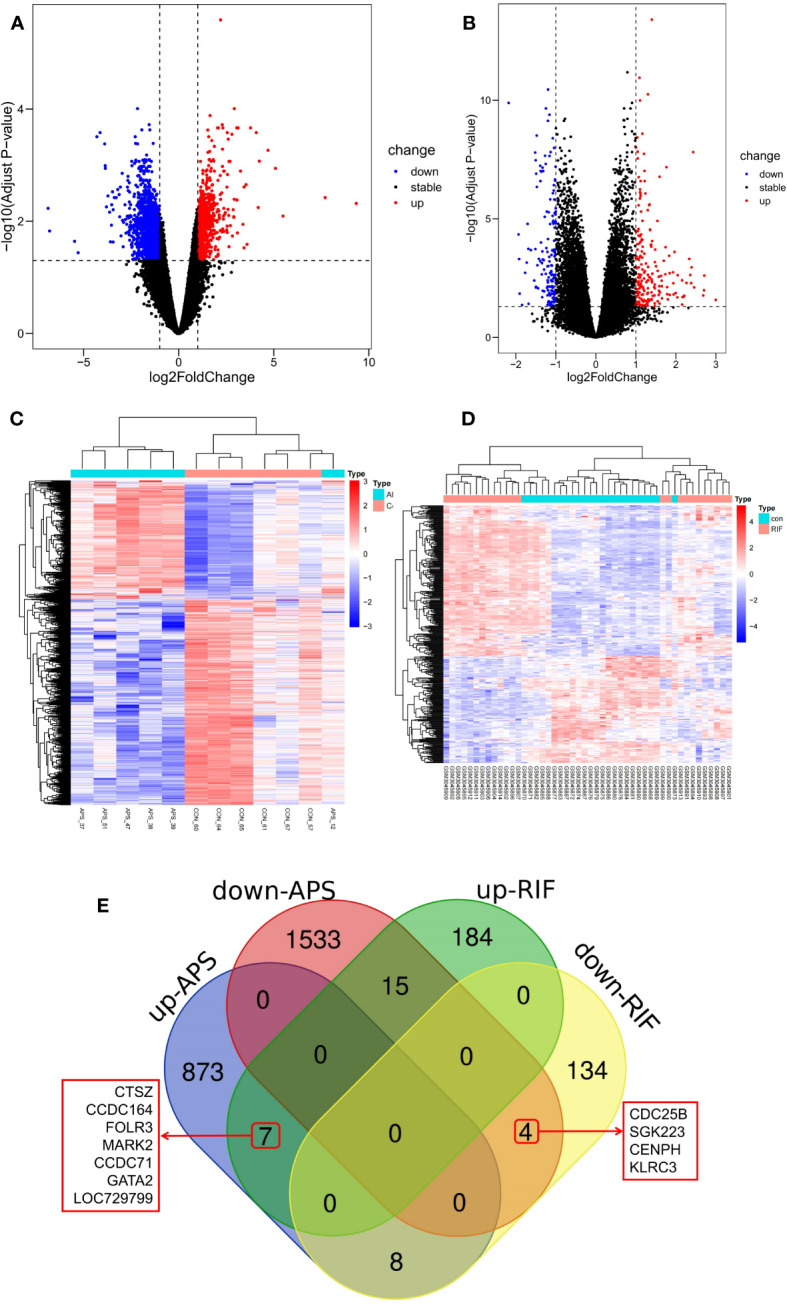
Screening of common differentially expressed genes (DEGs) between antiphospholipid syndrome (APS) and repeated implantation failure (RIF). **(A)** Volcano plots of APS DEGs. **(B)** Volcano plots of RIF DEGs. **(C)** Heatmap of the APS upregulated and downregulated genes. **(D)** Heatmap of the RIF upregulated and downregulated genes. **(E)** Venn diagram of the common DEGs in APS and RIF.

The DEGs of RIF and APS were obtained from the GSE102215 and GSE111974 datasets separately, with 888 upregulated and 1,552 downregulated genes in APS and 206 upregulated and 146 downregulated genes in RIF (**Figures 2A, B**). Heatmaps of the DEGs in both datasets are shown in **Figures 2C, D**. We identified the common DEGs from the intersection of the Venn diagrams (**Figure 2E**). After excluding genes with opposite expression trends, 11 common DEGs (seven common upregulated and four common downregulated genes) with the same expression trends were found in GSE102215 and GSE111974 (**Table 2**).

**Table 2:** The gene expression levels of 11 common differentially expressed genes (DEGs) in antiphospholipid syndrome (APS) and repeated implantation failure (RIF).

Gene samples	GSE 102215		GSE 111974		Up/Down
	logFC	P value	logFC	P value	
CTSZ	1.019004575	3.50E-06	1.0040625	1.07E-11	Up regulated
CCDC164	1.390055757	0.000210669	1.0521	0.001297453	Up regulated
FOLR3	1.259480744	0.042306007	1.412033333	0.000117374	Up regulated
MARK2	0.827205312	0.001059369	1.398616368	1.26E-18	Up regulated
CCDC71	1.001978252	0.000132575	1.2168125	2.48E-10	Up regulated
LOC729799	0.874322301	0.004536766	1.164225	1.49E-06	Up regulated
GATA2	1.51904247	0.00011236	1.150766667	6.79E-06	Up regulated
CDC25B	-1.254116899	0.000121245	-1.072204167	1.58E-07	Down regulated
SGK223	-1.119028202	0.000248632	-1.113441667	3.95E-05	Down regulated
CENPH	-1.186900689	0.007341801	-1.080745726	0.000316802	Down regulated
KLRC3	-1.937514717	1.60E-05	-1.093495833	0.000608419	Down regulated

### 3.3 PPI Network construction and enrichment analysis of common DEGs

**Figure 3:**
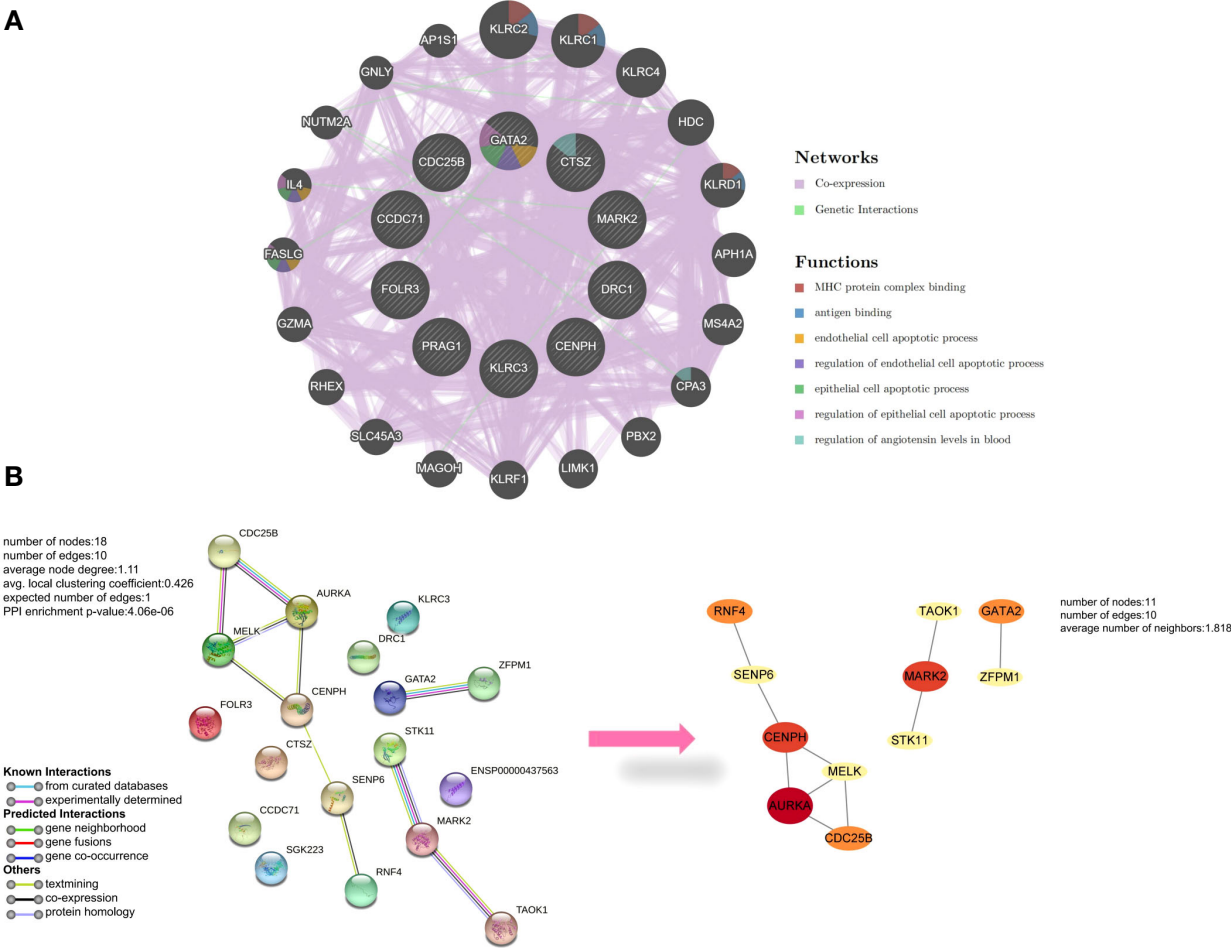
Analysis of common differentially expressed genes (DEGs) between antiphospholipid syndrome (APS) and repeated implantation failure (RIF). **(A)** Common DEGs and their co-expressed genes were analyzed using GeneMANIA. **(B)** The protein-protein interaction (PPI) network of the common DEGs.

With 99.92% co-expression and 0.08% genetic interactions, we created a sophisticated gene interaction network using the GeneMANIA database to understand the biological functions of these common DEGs. Twenty genes that were linked to the 10 common genes were identified, and the results revealed that they were mainly involved in MHC protein complex binding, antigen binding, endothelial cell apoptotic process, regulation of endothelial cell apoptotic process, epithelial cell apoptotic process, regulation of epithelial cell apoptotic process, and regulation of angiotensin levels in the blood (**Figure 3A**). According to the PPI network, AURKA, CENPH, and MARK2 had high degrees of number of connections to other points (**Figure 3B**).

**Figure 4:**
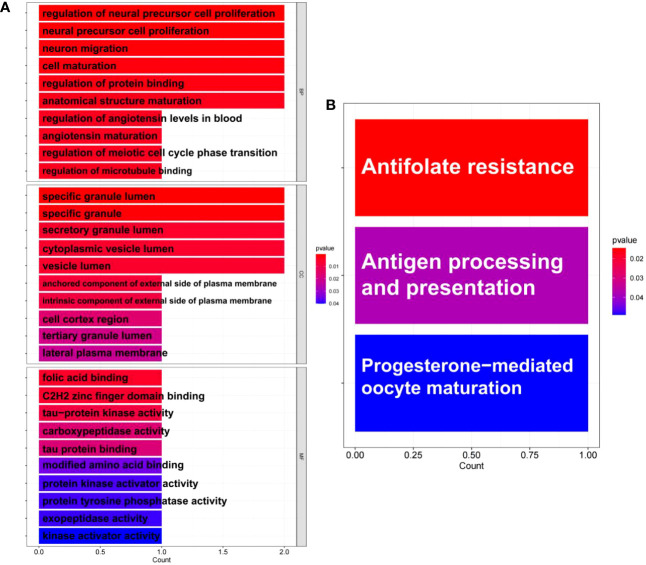
Enrichment analysis of common differentially expressed genes (DEGs) between antiphospholipid syndrome (APS) and repeated implantation failure RIF. **(A)** Gene Ontology (GO) enrichment analysis of the common targets. **(B)** Kyoto Encyclopedia of Genes and Genomes (KEGG) enrichment analysis of the common targets.

GO and KEGG pathway enrichment analyses were performed to further explore the biological roles of these common DEGs. For biological processes, the common DEGs were mainly related to the regulation of neural precursor cell proliferation, neural precursor cell proliferation, neuron migration, cell maturation, regulation of protein binding, and anatomical structure maturation. For cellular components, the common DEGs were mainly related to specific granule lumen, specific granule, secretory granule lumen, cytoplasmic vesicle lumen, and vesicle lumen. Finally, for molecular functions, the common DEGs were mainly associated with folic acid binding, C2H2 zinc finger domain binding, and tau-protein kinase activity (**Figure 4A**). Furthermore, KEGG analysis revealed that the common DEGs were mainly enriched in antifolate resistance, antigen processing and presentation, and progesterone-mediated oocyte maturation (**Figure 4B**).

### 3.4 The co-expression modules in RIF

As an autoimmune disease, the pathophysiology of APS is likely strongly related to the imbalance of immunological homeostasis *in vivo*. To further clarify if RIF is associated with the immune environment *in vivo*, we analyzed the key RIF genes.

**Figure 5:**
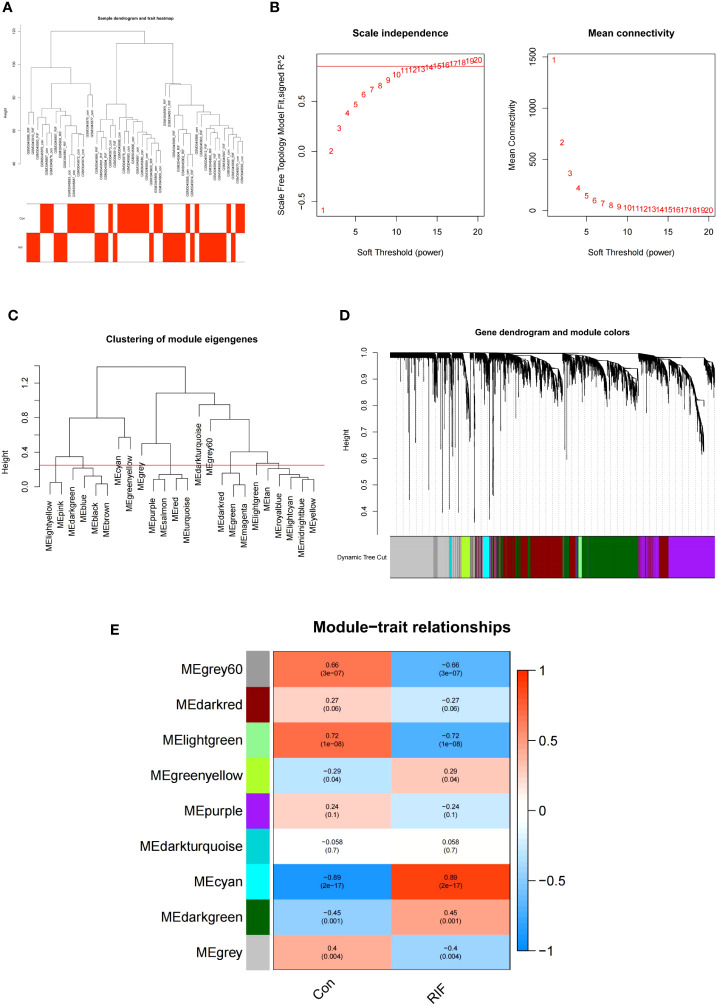
WGCNA of repeated implantation failure (RIF). **(A)** Sample clustering of RIF. The samples were classified into three significantly distinct clusters. All clusters were chosen for further analysis. **(B)** Selection of optimal thresholds. **(C)** The threshold was set to 0.25 to merge modules that are comparable in the cluster tree. **(D)** Different modules are produced and shown in different colors by aggregating genes with strong correlations into the same module. **(E)** Heatmap of the module-trait relationship in RIF. Each cell contains the corresponding correlation and *P*-value.

In the GSE111974 dataset, the gene that was significantly expressed (*P*<0.05) was selected as key genes of RIF for WGCNA analysis. All samples were clustered well and no sample was eliminated (**Figure 5A**). In our study, the optimal soft-power value of GSE111974 was β=15 (**Figures 5B, C**). A total of nine modules were identified. The correlations between modules and clinical diseases were then computed. The strongest positive association was seen in the cyan module (r = 0.89, *P* = 2e-17), while the light green module had the strongest negative correlation (r = -0.72, *P* = 1e-08) (**Figures 5D, E**). The list of genes in Co-expression Modules in RIF is given in **Supplementary Table 2**.

### 3.5 Screening and analysis of key genes in RIF

**Figure 6:**
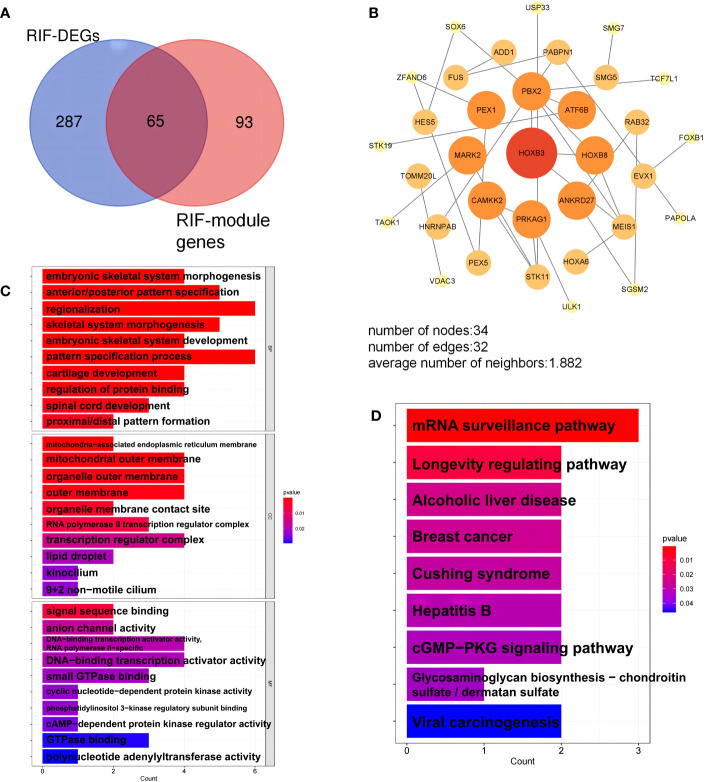
Analysis of key genes in repeated implantation failure (RIF). **(A)** The key genes are the overlapping differentially expressed genes (DEGs) and module genes. **(B)** The protein-protein interaction (PPI) network of the key genes. **(C)** Gene Ontology (GO) terms of biological process, cellular component, and molecular function were used for functional enrichment clustering analysis on key genes. **(D)** Kyoto Encyclopedia of Genes and Genomes (KEGG) pathway analysis was performed on key genes.

We selected the 65 overlapping genes in the DEG and module gene groups and labeled them as key genes **Figure 6A**, **Supplementary Data Table 1**). These genes were highly related to the pathogenesis of RIF. Moreover, the PPI network uncovered the close interactions between these common genes (**Figure 6B**). To investigate the potential functions of the key genes, we performed GO and KEGG pathway enrichment analyses. KEGG enrichment results showed that the mRNA surveillance pathway and longevity regulating pathway were significantly enriched (**Figure 6C**). As shown in the GO annotation results, the RIF key genes were markedly associated with embryonic development, pattern specification process, DNA-binding transcription activator activity, and RNA polymerase II-specific activator activity (**Figure 6D**).

### 3.6 Screening candidate diagnostic markers and developing a clinical predictive model

**Figure 7:**
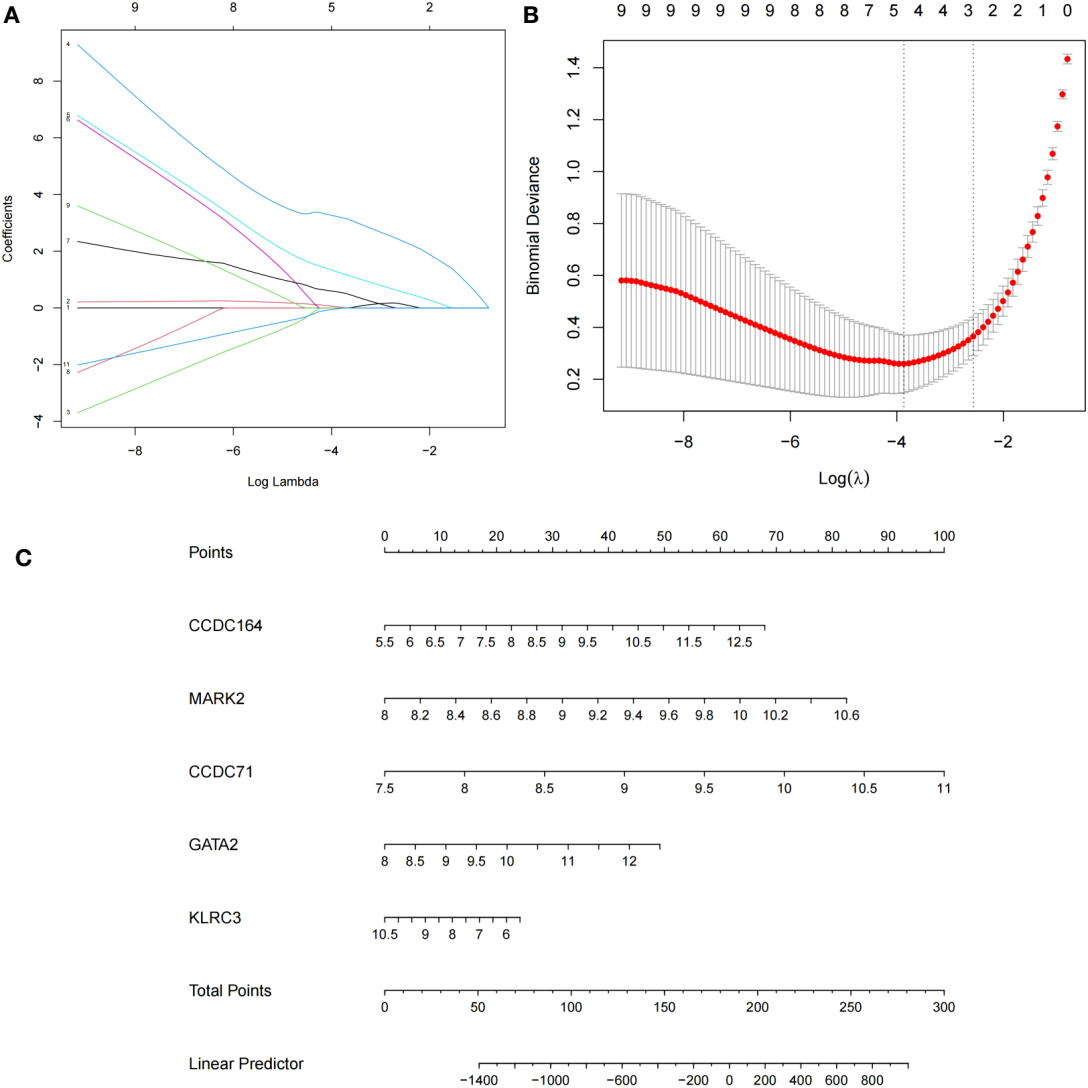
Establishment of the nomogram model of repeated implantation failure (RIF) with antiphospholipid syndrome (APS) and validation of these potential candidate biomarkers. **(A)** Tuning feature selection in the LASSO model. **(B)** LASSO regression coefficient profiles. **(C)** The nomogram for diagnosing RIF with APS.

Candidate genes were screened using LASSO regression in preparation for nomogram construction and diagnostic value assessment. Five probable candidate biomarkers (CCDC164, MARK2, CCDC71, GATA2, and KLRC3) were found via the LASSO regression algorithm (**Figures 7A, B**). The nomogram was then developed using these five candidate biomarkers (**Figure 7C**).

### 3.7 Validation of candidate diagnostic genes

**Figure 8:**
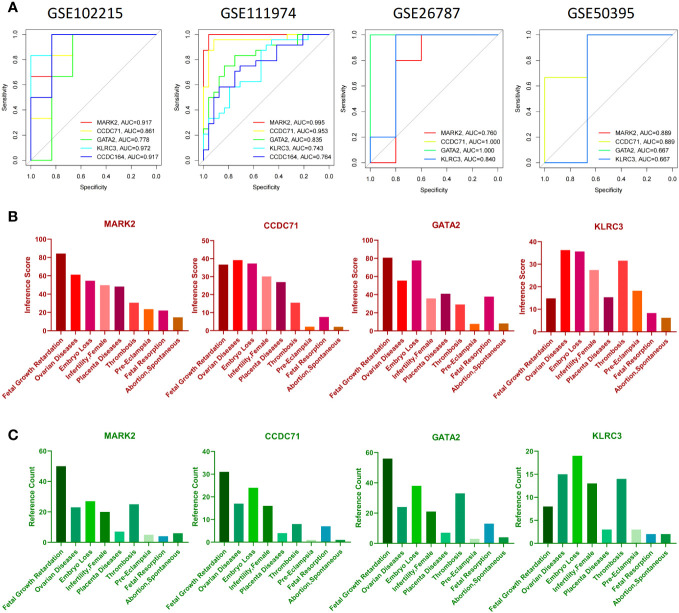
Verification of candidate diagnostic genes. **(A)** The receiver operating characteristic (ROC) curve of candidate diagnostic genes (MARK2, CCDC71, GATA2, and KLRC3) in GSE102215, GSE111974, GSE26787, and GSE50359. Inference score **(B)** and reference counts **(C)** between candidate diagnostic genes and fetal growth retardation, ovarian diseases, embryo loss, female infertility, placenta diseases, thrombosis, pre-eclampsia, fetal resorption, and spontaneous abortion in CTD.

To verify the reliability of these candidate diagnostic genes, we selected the GSE102215 and GSE50395 datasets to analyze their diagnostic efficacy in APS and the GSE11974 and GSE26787 datasets to analyze their diagnostic efficacy in RIF. Unfortunately, we retrieved only four candidate diagnostic genes in the validation cohort. Collectively, all four diagnostic genes had good diagnostic efficacy (**Figure 8A**). Furthermore, the data suggest that these four candidate diagnostic genes were not only associated with poor pregnancy outcomes in women but also with thrombosis (**Figures 8B, C**).

### 3.8 Immune cell infiltration analysis

**Figure 9:**
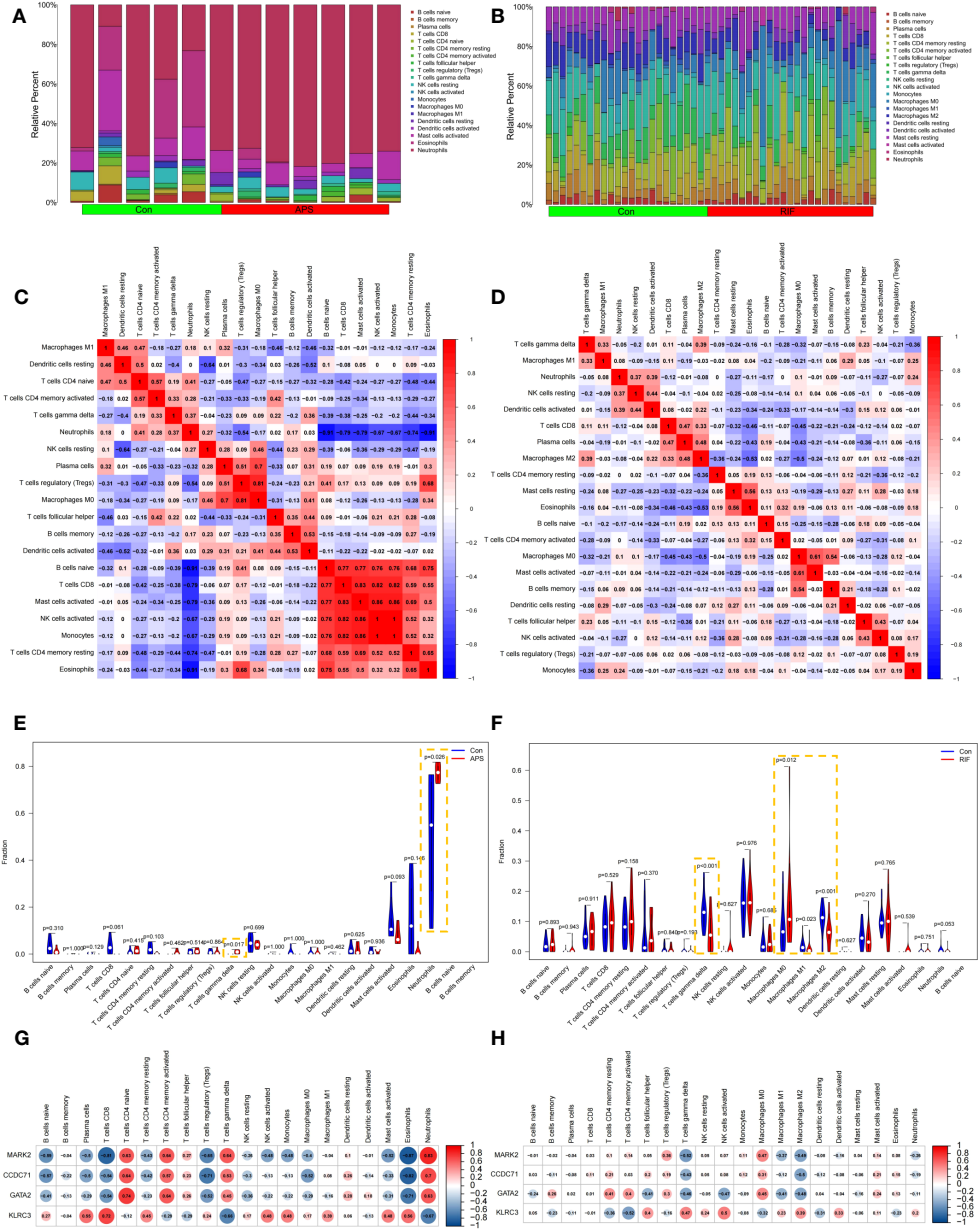
Immune infiltration analysis of antiphospholipid syndrome (APS) and repeated implantation failure (RIF). Bar graphs showing the immune infiltration of each sample in APS **(A)** and RIF **(B)**. Heatmaps showing the correlations between immune cells in APS **(C)** and RIF **(D)**. Violin plots showing comparisons of immune cells in APS **(E)** and RIF **(F)**. Correlations between immune cells and candidate diagnostic genes in APS **(G)** and RIF **(H)**.

Because we found that the common genes appear to regulate RIF pathogenesis via the immune system, we hypothesized that these genes may be used as prospective diagnostic biomarkers for RIF with APS using the nomogram and ROC analysis. To better clarify the immune regulation of RIF with APS, we examined immune cell infiltration. The bar graphs show that the percentage of neutrophils and T cell populations varied significantly between the APS and RIF samples (**Figures 9A, B**). Compared with the normal samples, neutrophils and γδ T cells were significantly increased in the APS samples (**Figure 9E**). However, in RIF samples, the M0 macrophage population was increased, while γδ T cells, M1 macrophages, and M2 macrophages were decreased (**Figure 9F**). **Figures 9C, D** depict the association between individual immune cells, demonstrating that neutrophils had a significantly negative correlation with other immune cell types in APS.

Furthermore, we investigated the correlations between candidate diagnostic genes and the levels of various immune cells. In APS samples, MARK2, CCDC71, and GATA2 had strong negative connections with CD8^+^ T cells and Treg populations. However, they had significant positive connections with both naive CD4^+^ T cell and neutrophil populations. KLRC3 significantly negatively correlated with T cells and neutrophils, and significantly positively correlated with CD8^+^ T cells (**Figure 9G**). In RIF samples, MARK2, CCDC71, and GATA2 had strong negative correlations with γδ T cells and M2 macrophages, but there was a positive correlation between KLRC3 and these two immune cells (**Figure 9H**).

### 3.9 Candidate drugs prediction

**Figure 10:**
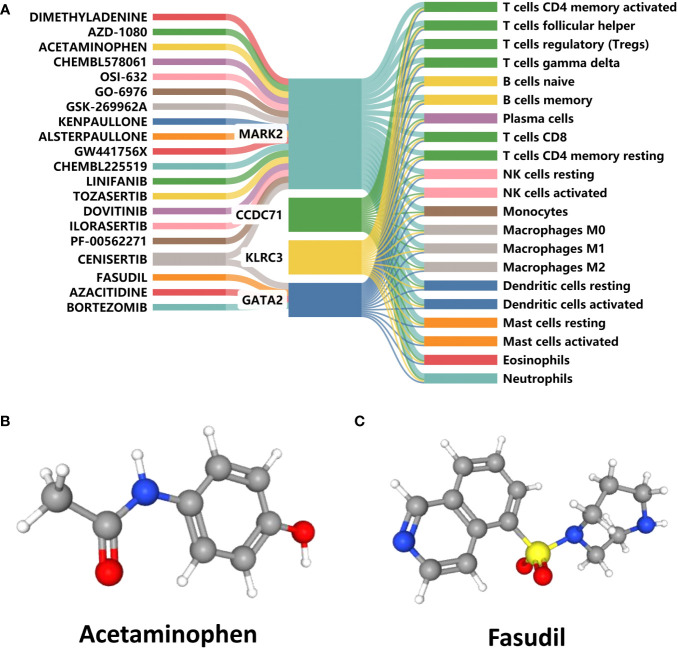
Prediction of candidate drugs. **(A)** Sankey diagram demonstrating the flow between candidate drugs, genes, and immune cells. **(B, C)** The 3D structure tomographs of the candidate drugs for repeated implantation failure (RIF) with antiphospholipid syndrome (APS).

The DGIdb was analyzed to identify small-molecule drugs with potential therapeutic effects on RIF with APS. The Sankey diagram was then used to demonstrate the interactions between small-molecule drugs, genes, and immune cells (**Figure 10A**). In contrast to CCDC71 and KLRC3, MARK2 and GATA2 had a relative abundance of targeted drugs and were associated with a variety of immune cells, which were important potential therapeutic targets for APS and RIF. Acetaminophen and Fasudil were predicted to be potential drugs for the treatment of RIF with APS. The 3D structure tomography of Acetaminophen (**Figure 10B**) and Fasudil (**Figure 10C**) was found in PubChem.

## 4 Discussion

This study was conducted to investigate the connection between APS and RIF using bioinformatics and machine learning approaches. Three upregulated genes (MARK2, CCDC71, GATA2) and one downregulated gene (KLRC3) were identified as candidate diagnostic genes that connect APS and RIF. In addition, γδ T cells were an important cause of APS and RIF. Enrichment analysis further indicated that multiple immune responses, including antifolate resistance, antigen processing, and presentation and progesterone-mediated oocyte maturation, were closely correlated with higher expression levels of common genes. According to CTD, the four candidate diagnostic genes have strong associations with female fertility, thrombosis, and ovarian diseases. As a result, we concluded that MARK2, CCDC71, GATA2, and KLRC3 may be crucial in immunological mechanisms during the development of APS and RIF.

As two significant disorders impacting the reproductive health of women, APS and RIF are closely related and intricately intertwined. There is accumulating evidence that the two diseases share multiple common risk factors, and APS may contribute to the pathogenesis of RIF (39). Recently, the maternal-fetal immune response has become an important trend in RIF pathogenesis (40). Immune cells are crucial for embryo implantation, immune tolerance, and embryonic growth throughout a healthy pregnancy. Immune disorders in APS patients cause an imbalance of intercellular and cell-secreted cytokines, which can lead to maternal rejection of the fetus and ultimately pathological pregnancy. When Chao et al. (41) treated APS mice with a mixture of DNA vaccine and FK506 adjuvant, they observed reduced Th1 and Th17 cell responsiveness and an increased frequency of Foxp3^+^CD4^+^Treg cells in splenic T cells after stimulation with β2-GP1. It is hypothesized that excessive Th1 and Th17 responses and reduced Treg levels are associated with the pathogenesis of APS. Excessive Th17 cell responses induce decidua natural killer (NK) cell activation and impair vascular reactivity of uterine arteries, leading to embryonic resorption (42). Wang et al. also suggested that IL-2 and TNF-α in recurrent miscarriage (RM) with APS peripheral circulation may be involved in the apoptosis of trophoblast cells and activation of NK cells, promoting the development of RPL with APS. Therefore, in RIF with APS, the imbalance of the maternal-fetal immunological microenvironment may be significant. These findings aid in illuminating the molecular mechanisms that connect APS and RIF. However, few researchers have explored the shared pathogenesis of APS and RIF at the genetic level. By combining data from multiple public databases, our study identified candidate diagnostic genes that can serve as biomarkers or potential therapeutic targets for APS and RIF, laying the foundation for determining common mechanisms of APS and RIF and possible clinical treatment methods.

In our study, we focused on four candidate genes to elucidate the relationship between APS and RIF. GATA2, a member of the GATA family of zinc-finger TFs, is classified with GATA1 and GATA3 as “hematopoietic” GATA factors that regulate the development of hematopoietic systems (43). GATA2 protein is crucial for controlling endometrial stromal cell decidualization (44), endothelial cell function and angiogenesis (45), adipocyte differentiation (46), and pituitary function (47). Rubel et al. (48) found that GATA2 is mainly expressed in the uterine luminal and glandular epithelium before embryo implantation and spatio-temporally co-localizes with the progesterone receptor. Mice with uterine-specific ablation of GATA2 displayed inadequate endometrial decidualization, implantation failure, and infertility. Furthermore, knockdown of GATA2 markedly decreased the expression of decidua markers in human endometrial stromal cells (49). According to these results, normal expression of GATA2 is important for the maintenance of endometrial decidua and support of normal embryonic development. In our study, we found that both APS and RIF had increased GATA2 expression levels. Because endometrial decidualization is a critical step in the acquisition of endometrial tolerance and successful embryo implantation, this demonstrates the close association between GATA2 and female pregnancy and confirms its role in APS and RIF. In addition, GATA2 serves as a positive regulator of the cell cycle by inducing the proliferation of macrophages and endothelial cells (50), which may be a reason for the observed increase in endometrial macrophages in patients with RIF.

In mammals, the MARK family consists of four members: MARK1−MARK4. MARK2 is a serine/threonine protein kinase involved in the phosphorylation of microtubule-associated proteins, such as Tau proteins, cell cycle-regulated phosphatases, and class IIa histone deacetylases, such as HDAC7. MARK2 is localized to the cell membrane and controls microtubule stability by phosphorylating microtubule-associated proteins (51), and engages in the regulation of mammalian immune homeostasis (52), fertility (53), and growth and metabolism (54). Although B and T cells developed normally in MARK-null mice, CD4^+^ T cells lacking MARK2 were expressed abnormally and produced more INF-γ and IL-4 upon stimulation through the T cell receptor *in vitro (* 52). In addition, the response of B cells when attacked by T cell antigens was altered *in vivo*. In addition, MARK2 plays a crucial role in the regulation of prolactin (PRL) secretion in female mice. Because PRL is essential for maintaining luteal function and progesterone secretion, PRL deficiency caused by MARK2 dysfunction may lead to infertility in females (53). Our research revealed that MARK2 expression was elevated in both APS and RIF. Previous studies that have addressed MARK2 expression have only focused on its inhibition. According to our research, the mammalian immune system’s stability depends on the MARK2 protein kinase, so aberrant expression could possibly result in autoimmune diseases and pregnancy complications.

CCDC71 and KLRC3 are also two important candidate genes identified in our study. KLRC3 is a member of the natural killer group (NKG) 2, which can specifically regulate humoral and cellular immunity. Although the role of KLRC3 in APS and RIF pathogenesis has not yet been investigated, it is associated with the development of other autoimmune diseases, metabolic diseases, and tumors (55–57). Downregulation of KLRC3 may lead to abnormal expression of the NKG2E receptor, which results in insufficient NK cell activation and defective NK cell function (56). In the mother, NK cells are involved in the invasion of trophoblast cells, remodeling of spiral arteries, secretion of cytokines, and regulation of the immunological balance of the maternal-fetal interface. Alterations in the quantity and function of NK cells, as well as imbalances between NK cells and other immune cells, may result in pathological pregnancies, including pre-eclampsia, RM, and RIF (58). Interestingly, a recent study suggests that insufficient rather than excessive uterine NK activation may contribute to the occurrence of RM and RIF (59), which further supports the impact of RIF with APS. CCDC71 protein, which has coiled-coil domains, is enriched in the nuclear periphery of HeLa and MCF7 cells and generates nuclear foci in U2OS cells (60). Currently, there are few studies on CCDC71 in APS and RIF. Therefore, the relationships between CCDC71, APS, and RIF deserve further exploration.

The results of KEGG analysis suggested that common DEGs play an important role in antifolate resistance, antigen processing and presentation, and progesterone-mediated oocyte maturation. Folic acid, as a one-carbon unit carrier, plays an important role in the prevention of birth defects and in preventing the development of adverse pregnancy outcomes (61). Folate deficiency causes an increase in DNA strand breaks and an increased incidence of errors during DNA replication (61), which in turn causes DNA hypomethylation and interferes with DNA synthesis and damage repair, resulting in DNA strand breaks, altered chromosomal recombination, and segregation abnormalities (62). Progesterone is closely related to the normal development of the embryo and has an important supportive role in early pregnancy. It has been proved by studies to be a specific marker of pregnancy and is of great value in the diagnosis of early pregnancy (63, 64). Our study found that common DEGs were strongly associated with antifolate resistance and progesterone-mediated oocyte maturation. However, further research is needed to determine whether and how much the common DEGs differ in expression levels among RIF patients.

The KEGG analysis results suggest that the immune disorder is the primary mechanism of APS and RIF. Immune cell changes are directly associated with immune homeostasis at the maternal-fetal interface. Abnormalities in immune cell quantity and function appear to significantly link APS and RIF. In our research, γδ T cells were lower in endometrial tissues in RIF than in normal tissues. These findings are consistent with the conclusions reached by Feng et al. (65) However, the number of γδ T cells was increased in the peripheral blood of APS patients. This may be a result of the different tissue types used to analyze the research participants (66), but our results still point to the significance of aberrant γδ T cells in the pathogenesis of APS and RIF. In subsequent work, the mechanisms by which γδ T cells affect endometrial tolerance also need to be further explored to provide more robust evidence for our current study.

Common DEGs are crucial and co-varied genes between APS and RIF, and their impacts on immune cells may be a potential mechanism for the link between APS and RIF. Our study demonstrated that MARK2, CCDC71, GATA2, and KLRC3 might affect the quantities of γδ T cells and macrophages. This implies that there are increased expression levels of candidate diagnostic genes in circulating immune cells in APS patients, with consequential changes in T cells and macrophages in the endometrium. This will disrupt maternal-fetal immune homeostasis and interfere with embryonic implantation (40), thereby increasing the risk of RIF or worsening RIF symptoms in patients with APS.

Finally, Acetaminophen and Fasudil were predicted as candidate drugs for the treatment of RIF with APS after reviewing the DGIdb database. Evidence suggested that Acetaminophen has negative immunomodulatory effects that can significantly increase the Treg cell population (67) and inhibit T cell-dependent antibody responses (68). Treg cells can assist with regulating maternal vascular function during pregnancy and support normal fetal and placental development, in addition to suppressing inflammation and preventing maternal over-immunity in response to the fetus (69). Through modifying the tolerance balance of T lymphocyte subsets at the maternal-fetal interface, Acetaminophen appears to contribute to the regulation of these maternal over-immune responses. Furthermore, Acetaminophen is less dangerous than other nonsteroidal antiinflammatory drugs because it does not produce severe gastrointestinal bleeding and has no impact on platelet function (70). However, the safe use of Acetaminophen during pregnancy remains a point of contention. Fasudil is another potential candidate drug identified in our study. It is a ras homolog-associated kinase inhibitor that participates in the regulation of T and B cells to achieve anti-inflammatory and immunomodulatory effects (71, 72). Excitingly, Fasudil has been shown to have beneficial effects in a range of cardiovascular and autoimmune diseases (73, 74). This suggests that perhaps Fasudil could play an unexpected role in the treatment of APS. These findings indicated that Acetaminophen and Fasudil might have effects on RIF with APS. In this study, maternal-fetal immune disorders are associated with the development of RIF, indicating that immunotherapy is a prospective treatment modality for RIF and APS. Although drug repurposing is an effective strategy to search for therapeutic candidates, due to these drugs and target genes are only experimental predictions, more evidence and data from extensive animal experiments and clinical trials need to be obtained.

### 4.1 Strengths and limitations

Our research addresses a gap in previous mechanistic studies and expands new ideas for future research. It is the first study to investigate the functions of common DEGs in APS and RIF and explore their link by bioinformatics. The application of new tools like machine learning and public databases makes our research more comprehensive and novel, and also makes the results more reliable. More importantly, our common DEG-based prediction model has demonstrated good predictive value. These genetic markers can provide higher specificity and sensitivity, especially when they are associated with specific disease subtypes or specific stages of disease progression.Certain genetic markers may be altered in the early or pre-disease stage, and can therefore be used as a tool for early diagnosis to avoid embryo implantation failures.Additionally, we visualized the prediction model, which can be used for clinical diagnosis and treatment. At the moment, the four candidate genes are only in the gene pool, there was no further analysis of the process of expression regulation of these genes, which is the focus of our next work. Because APS is a systemic disease that is limited by ethical and current experimental conditions, only datasets of peripheral blood samples can currently be retrieved, which may affect the results. As our research only used bioinformatics analysis with no animal or clinical experimental validation, further experimental research and clinical trials are needed to validate the biological functions of the candidate diagnostic genes. Furthermore, the safety and efficacy of candidate drugs *in vivo* also need to be verified.

## 5 Conclusion

In conclusion, through a series of bioinformatics analyses, we identified four key genes (MARK2, CCDC71, GATA2, and KLRC3) that could serve as biomarkers or potential therapeutic targets, and then established an effective diagnosis model. Meanwhile, the candidate drugs were predicted. These findings may provide a new and powerful scientific foundation for the diagnosis and treatment of RIF with APS.

## Data availability statement

The original contributions presented in the study are included in the article/**Supplementary materials**. Further inquiries can be directed to the corresponding author.

## Author contributions

XL and MZ conceived and designed the study. TG and MZ performed data analysis and interpretation. TG and YZ conducted bioinformatics analyses and created the figures and tables. MZ wrote the first draft, and XL and YZ conceptualized and revised the manuscript. All authors contributed to the article and approved the submitted version.
