# Supplementary material for: Identification of MARK2, CCDC71, GATA2, and KLRC3 as candidate diagnostic genes and potential therapeutic targets for repeated implantation failure with antiphospholipid syndrome by integrated bioinformatics analysis and machine learning
Source: Front Immunol. 2023 Oct 13;14:1126103. doi: 10.3389/fimmu.2023.1126103 (PMC10603295; doi:10.3389/fimmu.2023.1126103)
Supplement: Supplementary file 1 [file DataSheet_1.docx]

Supplementary Material

# Supplementary Data

**Supplementary table 1** The gene expression levels of 65 key genes in RIF.

Up-regulated genes

| Gene samples | logFC | P Value | UP/DOWN |
| --- | --- | --- | --- |
| MARK2 | 1.398616368 | 1.26E-18 | Up regulated |
| SMG5 | 1.090345833 | 1.07E-15 | Up regulated |
| PAPOLA | 1.302166667 | 8.83E-15 | Up regulated |
| USP33 | 1.104291667 | 1.91E-14 | Up regulated |
| EVX1 | 1.162650591 | 1.77E-12 | Up regulated |
| C19orf22 | 1.036285794 | 3.25E-12 | Up regulated |
| CTSZ | 1.0040625 | 1.07E-11 | Up regulated |
| ADD1 | 1.045325 | 1.85E-11 | Up regulated |
| PDZD8 | 1.202791667 | 2.62E-11 | Up regulated |
| PBX2 | 1.0473875 | 4.70E-11 | Up regulated |
| EHF | 1.764791667 | 1.43E-10 | Up regulated |
| XLOC_l2_007271 | 1.017029167 | 1.29E-05 | Up regulated |
| CCDC71 | 1.2168125 | 2.48E-10 | Up regulated |
| ZC3H7B | 1.5927875 | 5.39E-10 | Up regulated |
| MIR17HG | 1.072383333 | 5.70E-10 | Up regulated |
| HOXA6 | 1.221810054 | 9.08E-10 | Up regulated |
| HOXB3 | 1.104497659 | 2.79E-09 | Up regulated |
| DYTN | 1.105258333 | 4.06E-09 | Up regulated |
| FRYL | 1.239443593 | 4.73E-09 | Up regulated |
| FAM83B | 1.115954167 | 4.77E-09 | Up regulated |
| ATF6B | 1.100837268 | 8.69E-09 | Up regulated |
| GALNT10 | 1.341075635 | 1.92E-08 | Up regulated |
| HOXB8 | 1.333779167 | 2.10E-08 | Up regulated |
| SCARNA9 | 1.070045833 | 2.12E-08 | Up regulated |
| BDNF-AS1 | 1.131433333 | 4.59E-08 | Up regulated |
| XLOC_013475 | 1.167966667 | 1.33E-07 | Up regulated |
| SOX6 | 1.537661325 | 1.36E-07 | Up regulated |
| CAPS | 1.06875 | 3.79E-07 | Up regulated |
| C20orf96 | 1.352332326 | 4.15E-07 | Up regulated |
| TOMM20L | 1.044270006 | 5.10E-07 | Up regulated |
| TCF7L1 | 1.1298875 | 7.69E-07 | Up regulated |
| SNORD89 | 1.03775 | 2.69E-06 | Up regulated |
| CDC14A | 1.380629167 | 3.09E-06 | Up regulated |
| RNU105A | 1.114870269 | 1.20E-05 | Up regulated |
| XLOC_l2_001971 | 1.017029167 | 1.29E-05 | Up regulated |
| HES5 | 1.166287074 | 6.78E-05 | Up regulated |
| CLIC3 | 1.071508333 | 0.000327646 | Up regulated |
| TTC21A | 1.473825 | 0.000801993 | Up regulated |

Down-regulated genes

| Gene samples | logFC | P Value | UP/DOWN |
| --- | --- | --- | --- |
| XLOC_l2_015397 | -1.192708333 | 4.44E-15 | Down regulated |
| OSTCP2 | -1.246666667 | 6.90E-14 | Down regulated |
| PITHD1 | -1.196145833 | 4.18E-13 | Down regulated |
| VDAC3 | -1.075458333 | 3.64E-12 | Down regulated |
| GIMAP2 | -1.128984304 | 2.06E-11 | Down regulated |
| CTSL1P8 | -1.515128923 | 2.27E-11 | Down regulated |
| SUMO1P3 | -1.156833333 | 3.51E-11 | Down regulated |
| FOXB1 | -1.502375 | 5.84E-11 | Down regulated |
| LPCAT1 | -1.2517 | 9.89E-11 | Down regulated |
| XLOC_009080 | -1.314531142 | 2.54E-10 | Down regulated |
| FUS | -1.378516667 | 2.58E-10 | Down regulated |
| PRKAG1 | -1.360422322 | 2.88E-10 | Down regulated |
| LOC100289186 | -1.490558333 | 3.88E-10 | Down regulated |
| LOC100287063 | -1.0190875 | 7.48E-10 | Down regulated |
| XLOC_l2_000657 | -1.016725 | 8.42E-10 | Down regulated |
| XLOC_l2_001853 | -1.383777106 | 9.16E-10 | Down regulated |
| RAB32 | -1.0479625 | 1.62E-09 | Down regulated |
| 5-Mar | -1.036845833 | 9.93E-09 | Down regulated |
| LOC100127983 | -1.028473515 | 5.45E-08 | Down regulated |
| PEX5 | -1.005474658 | 6.30E-08 | Down regulated |
| QTRT1 | -1.002901564 | 1.49E-07 | Down regulated |
| CENPV | -1.002733333 | 1.78E-07 | Down regulated |
| STK19 | -1.144473843 | 2.04E-07 | Down regulated |
| HNRNPAB | -1.028537272 | 3.24E-07 | Down regulated |
| CHST11 | -1.2357375 | 4.41E-06 | Down regulated |
| SUPT16H | -1.051404167 | 6.67E-06 | Down regulated |
| SLC43A3 | -1.208979167 | 7.14E-06 | Down regulated |

**Supplementary table** 2 The list of genes in Co-Expression Modules in RIF.

| Module | Gene |
| --- | --- |
| cyan module | ZC3H7B,CTSL1P8,C20orf96,XLOC_l2_001853,FOXB1,HOXB8,MARK2,XLOC_l2_007147,XLOC_l2_007271,PAPOLA,FAM83B,EVX1,PRKAG1,HOXA6,CCDC71,XLOC_l2_015397,LPCAT1,XLOC_l2_001971,SCARNA9,DKFZp761E198,MIR17HG,QTRT1,LOC100289186,XLOC_009394,OSTCP2,JMJD1C,TMED9,PBX2,SUMO1P3,XLOC_009080,MARCH5,LOC285178,FLJ34208,ATF6B,XLOC_013475,LOC100507311,ANKRD36BP1,CTSZ,TRA2A,C19orf22,STK19,HIRA,SH3BP5,DPEP3,SLFN5,DYTN,MAT2B,LEO1,FAM18B2,USP33,ZNF486,LOC100287063,PITHD1,CENPT,DLGAP4,ANKRD36B,RNU105A,XLOC_l2_000657,SDK1,RBM47,LOC100505669,ADD1,LOC100130876,PLEKHG7,SMG5,NADKD1,TCF7L1,GPR135,UCP3,TSPAN5,YPEL4,FNIP1,XLOC_004050,XLOC_006814,MORC2-AS1, Q9N083, RIT1, IER3IP1, RGPD6, ALG9, KLHL28, LOC100506214,RAP2A,MAP4K3,LOC153684,SCAMP4,LOC284581,PUM2,SEMA6A,TMEM219,VDAC3,PEX5,FLJ11710,TOMM20L,GMEB2,SNORD89,OSR2 |
| light green module | TTC21A,EHF,CDC14A,HES5,CLIC3,CHST11,FRYL,GALNT10,SLC43A3,SOX6,CENPV,BAIAP2,PDZD8,CAPS,PDE3B,TMEM22,CAV2,LOC100506027,BDNF-AS1, MPP6, FUS,HNRNPAB,PNMA2,LOC100127983,PWWP2B,GIMAP2,RAB32,PRPS1L1,EBLN2,LOC100505504,SUPT16H,HHEX,CTSS,HOXB3,LMTK3,JAKMIP2,NCKAP1L,FOXK1,TCEAL5,NOXA1,MRAS,XLOC_011368,MMP15,LOC100129781,C12orf51,AKAP2,LOC100505500,SYTL1,TCERG1,MAML3,LOC100130000,SRF,CD93,LRRC37A2,XLOC_000393,FAM174A,INSIG2,TCEAL2,ADSL,C11orf61,NOS1 |

**Supplementary Figure 1**


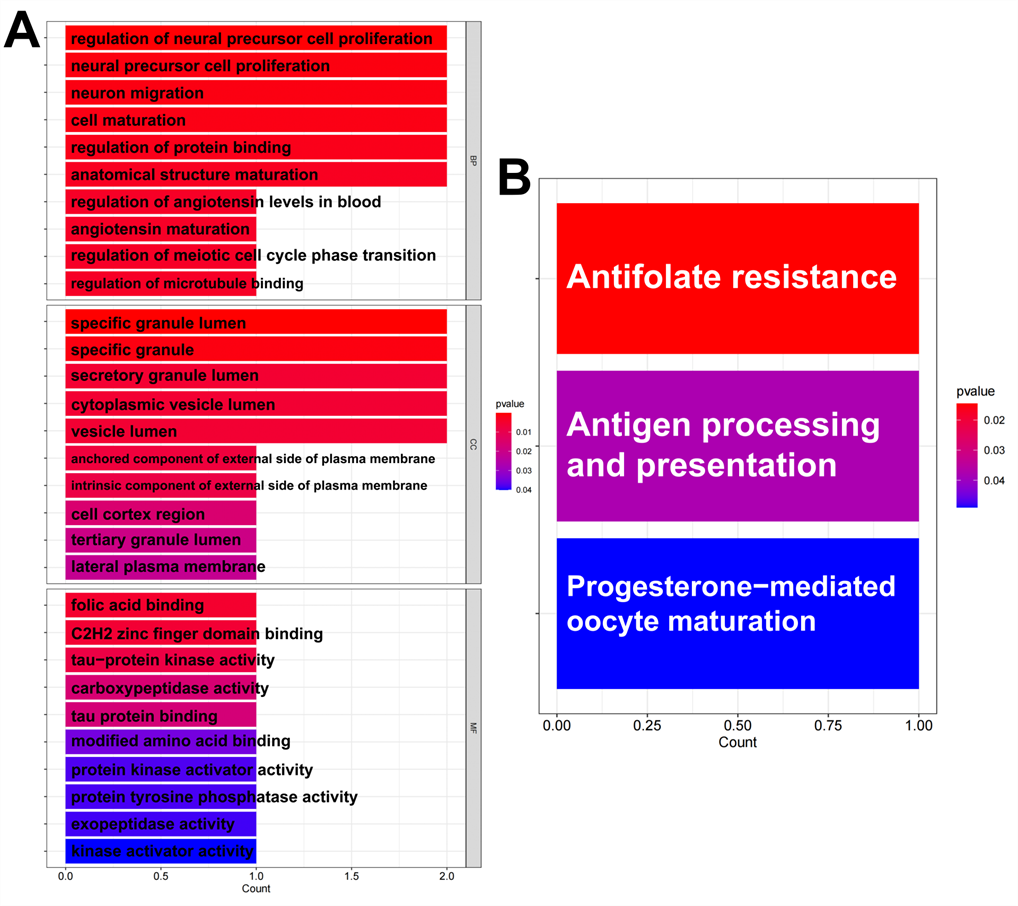


**Supplementary Figure 1:** Enrichment analysis of common differentially expressed genes (DEGs) between antiphospholipid syndrome (APS) and repeated implantation failure RIF. (A) Gene Ontology (GO) enrichment analysis of the common targets. (B) Kyoto Encyclopedia of Genes and Genomes **(**KEGG) enrichment analysis of the common targets.
